# Pelvic insufficiency fractures in the elderly – what insights has the past year of research brought? A narrative review

**DOI:** 10.1515/iss-2025-0018

**Published:** 2025-09-18

**Authors:** Kim Lydia Klepka, Christian Kleber, Yasmin Youssef

**Affiliations:** Waldklinken Eisenberg, Campus für Orthopädie, Medizinische Fakultät, Friedrich-Schiller-Universität Jena, 07607 Eisenberg, Germany; Klinik für Orthopädie, Unfallchirurgie und Plastische Chirurgie, Universitätsklinikum Leipzig, 04103 Leipzig, Germany

**Keywords:** pelvic insufficiency fracture, osteoporosis, diagnostic imaging, surgical treatment, pharmaceutical treatment, outcome

## Abstract

Pelvic insufficiency fractures (PIFs) are low-energy fractures typically occurring in osteoporotic bone and are associated with significant morbidity, reduced quality of life, and increased mortality. PIF requires individualized, often interdisciplinary treatment strategies combining surgical, conservative, and systemic approaches. This narrative review aims to summarize recent findings on the diagnosis, management, and outcomes of PIFs and place them in the context of existing literature. For this purpose, a selective literature search was conducted in January 2025 to identify publications on the topic of PIFs. The search was performed on PubMed and clinical studies addressing the diagnosis, treatment, outcomes and complications of PIFs from January 2024 to 2025 were selected for evaluation. A total of 11 studies with a total of 27,672 patients were included in this review. The findings emphasise the significant morbidity, mortality and healthcare burden associated with these fractures. Conventional radiography frequently fails to detect this type of fracture, thus necessitating advanced imaging techniques such as computed tomography (CT) and magnetic resonance imaging (MRI) to ensure an early diagnosis. The treatment decision is determined by the stability of the fracture, presence and intensity of pain, and the patient’s mobility levels. The treatment options, whether conservative or surgical, are adapted to these factors. The osteoporotic fracture (OF) Pelvis Score has emerged as a promising tool for guiding therapeutic strategies, though there is still potential for further development in the use of pharmaceutical treatment of underlying conditions such as osteoporosis. Despite the advances that have been made, the existing literature remains heterogeneous, underscoring the importance of conducting prospective studies and developing evidence-based guidelines. Interdisciplinary and orthogeriatric care is important for improving outcomes for this vulnerable patient group.

## Introduction

Pelvic insufficiency fractures (PIFs) are pelvic fractures that occur without trauma or due to low-energy trauma mechanisms like ground-level falls in osteoporotic bone. Within the German population, the incidence of PIFs is recorded as 22.4 per 10,000 individuals over the age of 60 and in the Netherlands, there was an increase in PIFs from 5.19 cases per 10,000 in 1986 to 7.17 cases per 10,000 in 2011 [1], 2]. So as the population ages, the incidence of PIF has increased significantly over the past decades and further increase is predicted [3]. PIFs represent a manifestation of systemic diseases that are commonly associated with impaired bone quality. PIF has been shown to be higher among women, due to hormonal changes after menopause and therefore higher rates of osteoporosis and lower bone mineral density, which increase bone fragility and susceptibility to insufficiency fractures [4]. These fractures have shown to be life-changing experiences for elderly patients as they result in pain, immobility or impaired mobility, and chronic instability. This causes a significant reduction of independence and therefore quality of life [5]. Furthermore, patients with PIF show an increased mortality. A current study has shown that the one-year mortality after osteoporotic pelvic ring fractures is over 20 % [6].

PIF differ greatly from pelvic ring fractures in young patients with respect to the injury mechanisms, clinical presentation and the treatment strategy. The occurrence of pelvic injuries in younger patients (aged between 20 and 30 years) is often associated with traffic accidents or falls from considerable heights, involving high-energy impacts. In polytraumatized patients, the incidence of pelvic injuries is 15 % and in these cases where multiple injuries require treatment, emergency care algorithms are used to guide it [7]. The treatment of PIFs includes both conservative and surgical approaches combined with systemic therapy and depends on the fracture type and stability of the fracture [8]. However, an interdisciplinary approach including orthopaedic surgeons, geriatricians, physiotherapists, and pain specialists is crucial to ensure optimal patient outcomes. Given the complexity of these fractures and the frequent presence of comorbidities in elderly patients, early collaboration is essential to balance surgical stabilization, pain management, and mobilization strategies. A coordinated effort between medical, surgical, and rehabilitation teams helps to reduce complications, improve functional recovery, and enhance overall quality of life [9].

The aim of this narrative review is to critically summarise and present the latest findings published between January 2024 and January 2025 on the diagnosis, treatment, complications, and outcomes of PIFs in older people over the age of 60. By focusing on this defined time frame and patient cohort, the aim is to identify current trends, therapeutic developments, and diagnostic advances. Furthermore, ongoing challenges and heterogeneity in clinical practice will be highlighted. The results will serve as a basis for future research directions, support the development of standardised treatment pathways, and serve as a foundation for possible systematic reviews or the development of guidelines. The clinical implications of this study may include the development of more targeted imaging strategies, the integration of interdisciplinary care models, and the improved implementation of pharmacological osteoporosis treatment in this vulnerable population.

## Methods

A selective literature research was carried out of all studies published on pelvic insufficiency fractures between 01/2024 and 01/2025 on PubMed. The search-terms “geriatric pelvic fractures”, “pelvic insufficiency fractures” and (pelvic fractures[Title/Abstract]) AND (elderly[Title/Abstract]) were used. A total of 114 articles were found. The titles of the 114 retrieved articles were then screened by two authors to identify relevant studies. The titles were examined according to the following criteria: the type of publication (case reports, case series, opinion papers, technical notes, scoping and systematic reviews and biomechanical studies were excluded), the language of publication (publications not written in German or English were excluded), and the topics covered (studies that did not address geriatric insufficiency fractures with a population older than 60 years and studies that did not deal with diagnostics, treatment, outcomes, or complications of PFIs were excluded). Articles that have not been published in peer-reviewed journals and duplicate studies were also excluded from the analysis. Subsequently, a comprehensive evaluation of the abstracts of the 31 remaining papers was performed, with relevant information being extracted from each study. This information included authors, publication year, study design, study population, location, main endpoint and main findings. Studies that dealt with other insufficiency fractures, such as acetabular or radius fractures, were excluded from the review. Studies that did not use a clear fracture classification and only referred to sacral fractures were also excluded from the analysis. The findings were organised in an Excel spreadsheet (Microsoft Corporation, USA) and discrepancies were resolved through discussion and consensus of all authors. Following a detailed evaluation, a total of 11 studies were selected for inclusion in this narrative review.

## Results

**Figure 1:**
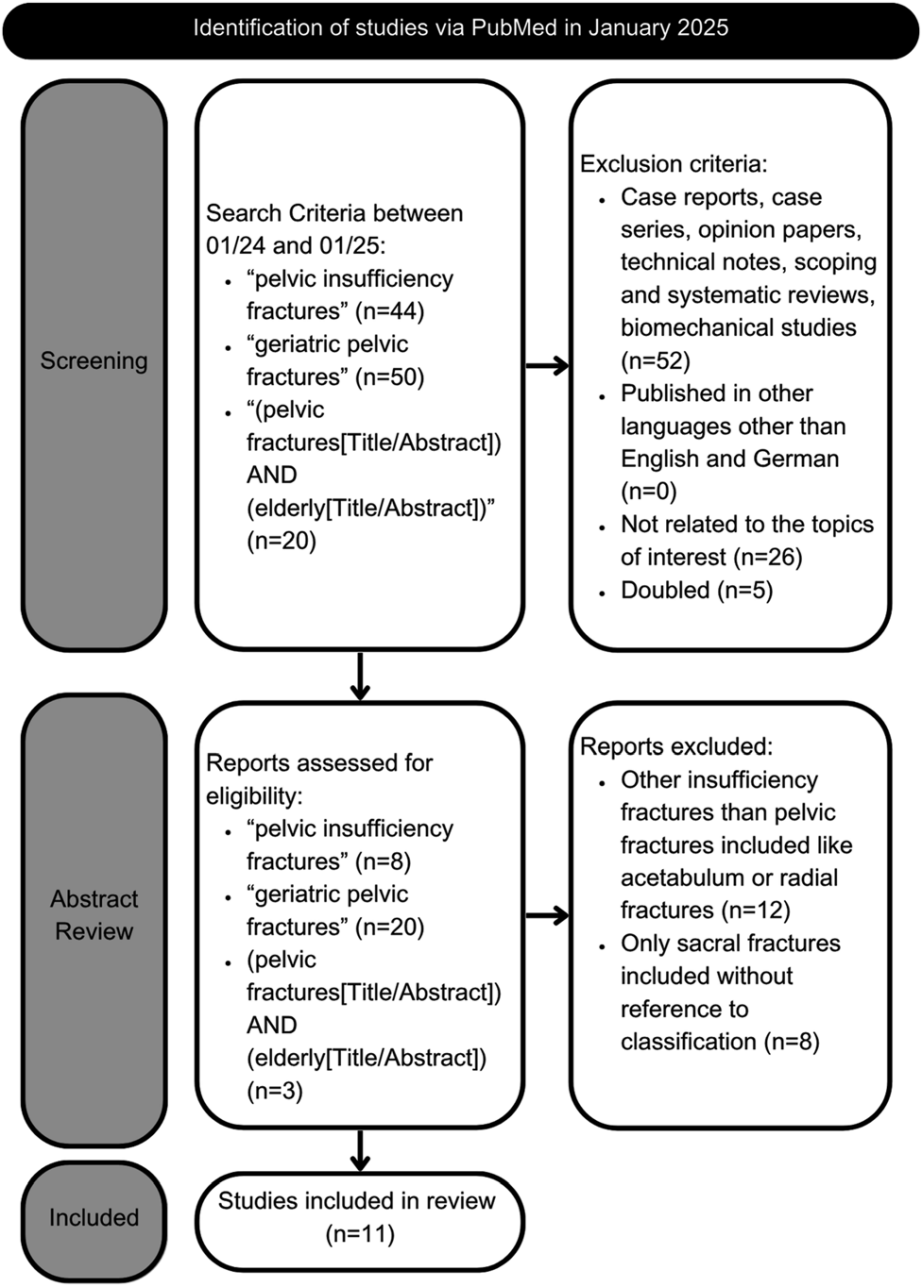
Flow chart of the literature search.

The result of the literature search is depicted in Figure 1. Of the 11 studies that were included in the review, one study focused on the diagnostic process of PFI, with a sample size of 107 patients [10]. Four studies concentrated on treatment, with a total of 1,338 patients included [11], [12], [13], [14]. A total of 1,358 patients were included in three studies that investigated the complications of PFI [15], [16], [17]. Three studies concentrated on the outcome and risk factors of insufficiency fractures of the pelvis and included a total of 24,869 patients [18], [19], [20]. The aggregate number of patients examined in these 11 studies was 27,672.

### Diagnostics

Early detection of PIF is crucial for initiating the appropriate treatment and reducing complications. Abdalmaqsoud et al. performed a retrospective cohort study including 107 patients (78.5 % female, mean age 83.07 years) from a Level 1 trauma centre, which aimed to assess the sensitivity and specificity of AP pelvic radiographs in patients aged>70 years with immobilizing pelvic pain [10]. The study assessed the diagnostic accuracy of anteroposterior (AP) pelvic radiographs in detecting pelvic ring fractures, using computed tomography (CT) as the gold standard. The findings revealed that AP pelvic radiographs had a remarkably low sensitivity of only 3.4 %, indicating a high likelihood of missing true fractures. However, specificity was relatively high at 94.4 %, meaning that most cases identified as negative were correctly classified. The positive predictive value (PPV) was 66.7 %, suggesting that when a fracture was detected on radiographs, it was confirmed by CT in about two-thirds of cases. In contrast, the negative predictive value (NPV) was only 23 %, highlighting that a negative radiograph result did not reliably exclude the presence of a fracture. The overall diagnostic accuracy of AP radiographs for pelvic ring fractures was limited, with an accuracy rate of just 24.7 %. Additionally, the study reported a significant delay in obtaining a definitive diagnosis after initial radiographic assessment, underscoring the need for improved imaging strategies to enhance early and accurate detection of pelvic ring fractures.

### Treatment

The goal of PIF treatment is to restore the patient’s mobility and independence as quickly as possible. As a tool to facilitate clinical decision making for choosing adequate treatment options for patients with PIF, Spiegl et al. developed the osteoporotic fracture (OF) Pelvis Score (Table 1), which is based on the OF-pelvis classification [13], 21]. In a retrospective cohort study, they screened all cases of osteoporotic pelvic ring fractures treated in three study centres between January 2020 and June 2020. Inclusion criteria included fracture without sufficient trauma, age over 65 years, and complete imaging (X-ray, computed tomography (CT), magnetic resonance imaging (MRI)). A total of 107 cases could be included. In the study, the OF Pelvis Score was retrospectively determined and examined to see if it corresponded to the therapy recommendation made individually at the time. From the 107 cases examined, 72 (67.2 %) exhibited a score below 8, thus indicating a recommendation for conservative treatment. Of these cases, 67 were treated conservatively, which corresponds to an agreement of 93 %. Conversely, 35 cases (37.5 %) exhibited a score of eight or above. Of these, 30 cases (32.1 %) were treated surgically. Four patients who were recommended to undergo surgery declined the operation. Excluding these cases, there was a retrospective agreement of the OF Pelvis scores of 94 %.

**Table 1:** OF Pelvis Score [13].

Characteristic	Severity	Points
Fracture morphology	OF pelvis classification: OF 1–5 x Factor 2	2–10
Mobilization	Immobile Very limited/previously immobile Sufficiently mobile	2 0 −2
Pain, VAS^a^	>5 <5	1 −1
Neurology	Fracture-related neurological deficit	1 if present
State of health	ASA^b^>3; mFI^c^>2 Anticoagulant medication	−1 each; max. −2
Modifying factors	M1=Transverse fracture on CT M2=Fracture dislocation M3=Fracture edema at an additional location on MRI	1 each, max. 1
Treatment recommendation	Conservative treatment Relative indication for surgery Surgical treatment	0–7 points 8 points 9–15 points

If a characteristic cannot be evaluated or is unknown, it is given 0 points. ^a^VAS, visual analogue scale; ^b^ASA-Score, American Society of Anaesthesiologists physical status classification; ^c^mFI, modified Frailty Index.

In cases where conservative treatment with pain-adapted mobilization and analgesic therapy does not produce the anticipated results, surgical intervention should be considered [7]. Swenson et al. demonstrated that percutaneous fracture treatment (iliosacral screw (SI-Screws) and/or transsacral–transiliac screw placement), with anterior fixation, if necessary, can provide significant pain relief for patients with failed conservative treatment [11]. In the retrospective examination of 231 patients, it was observed that 26.8 % of individuals underwent surgical intervention after unsuccessful mobilization attempts. These patients were significantly younger, had fewer pre-existing medical conditions, and had higher pain levels at admission to the hospital. Following treatment, there was a statistically significant decrease in pain in both the operative and nonoperative groups. Patients who underwent surgery had significantly longer inpatient stays. Otherwise, there were no significant differences between the two groups in terms of secondary endpoints such as treatment complications, 90-day mortality and 90-day readmission. However, a subsequent analysis of the subgroups revealed that additional anterior fixation of ramus pubis fractures demonstrated no statistically significant difference on the observed postoperative pain levels.

Similarly, Lampert et al. demonstrated an advantage for isolated, minimal-invasive posterior treatment in terms of length of hospital stay, even for fractures classified as unstable according to the fractures of the pelvis (FFP) classification [12]. In their retrospective study, they examined 882 patients over the age of 65 who were treated for a PIF in two trauma centres of differing sizes. Of these patients, 455 were treated at a level I trauma centre between 2003 and 2019, and 427 were treated at a level III trauma centre between 2011 and 2020. The study also compared the various surgical strategies, considering the severity of the fracture, in relation to length of stay. There was also a wide variance in the surgical methods used to treat the different types of fractures (Table 2).

**Table 2:** Surgical procedures used to treat the anterior and posterior pelvic ring [12].

Posterior pelvic ring	Anterior pelvic ring
– SI-Screw^a^ unilateral (1 level) – SI-Screw^a^ bilateral (1 level) – SI-Screw^a^ unilateral (2 levels) – SI-Screw^a^ bilateral (2 levels) – Lumbopelvic stabilization – Lumbopelvic stabilization + SI-Screw^a^ – Plate osteosynthesis – Internal fixator – Internal fixator + SI-Screw^a^	– IlluminOss – Plate osetosynthesis – IlluminOss + plate osteosynthesis – External fixator – External fixator + plate osteosynthesis – Creeping screw – Internal fixator

^a^SI-Screw, iliosacral screw.

Pharmaceutical interventions for the treatment of PIF have also been demonstrated to be an effective intervention, particularly parathyroid hormone (PTH) [22], 23]. Novikov et al. investigated the implementation of PTH-therapy in women over 60 with proven PIF [14]. In their retrospective cohort study, they evaluated the rate of PTH-supplementation following injury in 163 cases, included between 2012 and 2021, of stable pelvic fractures in female patients over 60 years. There was a large discrepancy between the medical specialties that recommended and initiated osteoporosis therapy. In 100 % of the patients, a PTH-therapy-evaluation was recommended by the orthopaedic service, and in 73.9 % of the cases the recommendation was documented in the initial consultation. In contrast, just 18 patients were evaluated for PTH-therapy by other medical specialties such as geriatricians, endocrinologists, or internists (17 %). Other specialties only prescribed PTH therapy in 9.6 % of the cases and started the therapy in 6.7 % of the cases.

### Complications

Patients with PIF face several complications like immobility, persisting pain, and adverse events like urinary tract infection and pneumonia, which can lead to loss of autonomy and higher mortality rates [5], 24]. In contrast to pelvic fractures resulting from injuries with a high-energy mechanism, where bleeding is known to be a significant and fatal complication, the possibility of bleeding as a complication also exists in PIFs with a low-energy mechanism [7], 25], 26]. McDonald et al. retrospectively analysed 457 cases of patients older than 65, over a period from 2010 to 2019, with a pelvic ring injury [16]. They assessed the prevalence of pelvic CT angiography (CTA) and the need for arterial embolization. The study compared patients with high- and low-energy injury mechanisms. A total of 91.4 % of the patients had a low-energy mechanism and among all 457 patients, 33 (7.2 %) received a pelvic CTA. Patients with high energy injuries were more likely to receive a pelvic CTA (p=0.0067), but this mechanism was not significantly associated with the need for an embolization (p=0.658).

Jammal et al. investigated the prevalence of severe hemorrhage in elderly patients aged over 65 with isolated and stable low-energy pelvic ring fractures [15]. They conducted a retrospective cohort study with 123 patients between January 2006 and December 2020. Significant blood loss was defined as a decrease in hemoglobin (Hb) of more than 2 g/dL in consecutive controls. In this cohort 24.4 % of the patients experienced a significant Hb drop, 15.5 % needed blood transfusions and 4 % interventions like arterial embolization. In addition, the influence of anticoagulant therapy on the risk of bleeding was investigated. It is noteworthy that 70 % of the bleeding cases were associated with posterior pelvic ring injuries, particularly in patients with anticoagulant therapy (p<0.01).

In contrast to this de Herdt et al. demonstrated that the risk of clinically significant bleeding is low for patients with a PFI after a low-energy trauma [17]. A total of 322 cases were evaluated retrospectively between January 2018 and August 2022. The primary outcome measure was the prevalence of a clinically relevant bleeding, which was defined as a bleeding that required blood transfusion or invasive treatment. In this cohort no invasive treatment had to be performed and just three patients required blood transfusions (transfusion rate: 0.9 %). However, it must be noted that these patients had a low Hb value on admission without hemodynamic instability and one had an additional diagnosed iron deficiency anaemia.

### Outcome and risk factors

The outcome of patients suffering from PIFs varies, with several factors influencing their prognosis. 1-year mortality after a PFI is substantial with rates from 10 to 27.3 % [27]. There is evidence that pre-injury functional status influences the outcome [28]. The role of frailty as a factor influencing functional status is frequently discussed [29]. Lo et al. analysed the impact of frailty on the short-term outcome of patients over 60 with a low-energy pelvic fracture [18]. In their retrospective observational study 24,688 cases, accessed from the American Nationwide Inpatient Sample (NIS) between 2016 and 2018, were evaluated to assess the impact of the modified Frailty Index (mFI-11). Patients with pelvic fractures after a high-energy injury and fractures besides the PFI were excluded. The groups were divided into frail and non-frail patients. The endpoints assessed were hospital mortality, unfavorable discharge, prolonged hospitalization, and complications. Findings revealed that 35 % of the patients were frail, and frailty was significantly linked to adverse outcomes. Frailty increased the odds of unfavourable discharge (adjusted odds ratio (aOR)=1.07), prolonged hospitalization (aOR=1.51), complications (aOR=1.42), and acute kidney injury (aOR=1.68). Stratified analyses indicated frailty consistently correlated with worse outcomes across age groups and fracture types.

Mair et al. investigated the prevalence and impact of sarcopenia and osteoporosis in 140 elderly patients with PIF [20]. Sarcopenia was defined as “a progressive and generalized skeletal muscle disorder that is associated with increased likelihood of adverse outcomes including falls, fractures, physical disability and mortality” and was assessed by evaluating psoas muscle area and the height adjusted psoas muscle index in CT scans. Sarcopenia was diagnosed in more than two thirds of the patients (68.6 %). Osteoporosis was diagnosed in 73.6 % of the patients. Patients who were diagnosed with both conditions experienced longer hospital stays, higher complication rates, and impaired functional recovery but were less likely to undergo surgery. Despite these findings, there was no significant difference in mortality, age, or gender. The study underscores the importance of addressing sarcopenia and osteoporosis to decrease the risk for (recurrent) PIF and improve recovery in elderly patients.

It is also hypothesised that fracture type, treatment approach, and pharmaceutical treatment may influence the outcome of patients with PFIs [27], 30], 31]. Bal et al. retrospectively investigated the outcome of 41 elderly patients with a low-energy pelvic fracture (pubic rami with involvement of the posterior pelvic ring) that were treated conservatively between January 2016 and December 2020, in a Swiss level 1 trauma centre [19]. The findings indicate that 78 % regained mobility, in terms of independent walking with or without orthopedic walking aids, within 2 weeks. Male sex identified as the sole predictor of non-mobility after 2 weeks (p=0.0037). Fracture consolidation occurred in 73.2 % of cases. Notably, displacement of over 5 mm did not affect mobility outcomes.

## Discussion

This study highlights key findings on pelvic insufficiency fractures in the elderly. Conventional radiographs show low sensitivity, underscoring the value of early CT or MRI for diagnosis [10]. Treatment depends on fracture stability, pain, and mobility; while stable fractures are treated conservatively, selected cases benefit from minimally invasive surgery [11], 12]. The OF Pelvis Score shows potential for guiding therapy but requires further validation [13]. Pharmaceutical treatment, especially osteoporosis management, remains underused [14]. Comorbidities such as frailty and sarcopenia significantly impact outcomes and support the need for orthogeriatric co-management. [18], [19], [20]. The findings of this narrative review highlight the significant burden of PIF in an aging population, underscoring the need for optimized diagnostic and treatment strategies. PIF have been rising in incidence in the past years, especially due to aging populations in the western world [1], 3]. PIF are associated with high morbidity and mortality, as well as with high healthcare costs [27], 32], 33]. An interdisciplinary approach is highly recommended in the treatment and follow-up treatment of these patients [9]. Despite the efforts to establish diagnostic and treatment algorithms literature remains heterogenous and big prospective and multicentre studies are warranted. Above all, there is a lack of established models for systemic therapy to treat underlying diseases, especially osteoporosis.

The studies included in this review confirm that PIFs are frequently underdiagnosed due to limitations of conventional radiographs and often require advanced imaging modalities, such as CT or MRI, for accurate detection [10]. The clinical presentation is most often immobilizing pain in the pelvic region and/or lower back after no or low-energy trauma. MRI is of significant value in the early phase of a PFI. It can detect bone edema on the ipsi- or contra-lateral side, a factor that has the potential to influence the therapeutic approach prematurely [34]. It has been shown that PIF are often misclassified or even missed when only using conventional radiographs as a diagnostic tool. Scheyerer et al. [35] found that in 96.8 % of their examined cases, pubic rami fractures were accompanied by posterior pelvic ring fractures that were not initially visible on standard pelvic radiographs but were later detected using CT imaging. Wagner et al. [36] recommended a diagnostic algorithm for detecting fragility fractures of the sacrum. In this algorithm conventional radiographs of the pelvis are recommended if there is a clinically suspected. A CT-scan is then recommended according to the findings in the radiograph or when pain persists. The diagnostic approach was then tested and validated by Beelen et al. [37] The study showed that performing a CT only had a limited direct therapeutic consequence. In addition to that the study found that sacroiliac joint (SIJ) pain has been shown to have a sensitivity of 89 % and a specificity of 61 % in detecting concomitant pelvic ring fractures. While this suggests that SIJ pain is a strong indicator of underlying fractures, the moderate specificity indicates that not all cases of SIJ pain are associated with pelvic ring injuries. Given this diagnostic accuracy, it has been proposed that an additional CT scan should be reserved for patients presenting with SIJ pain, rather than performing CT imaging routinely. Similarly, Abdalmaqsoud et al. [10] conducted a retrospective cohort study on 107 elderly patients with immobilizing pelvic pain, evaluating the diagnostic accuracy of AP pelvic radiographs for detecting pelvic ring fractures. The study found a very low sensitivity (3.4 %) but high specificity (94.4 %), indicating that while true fractures were often missed, most negative cases were correctly classified, emphasizing the need for improved imaging strategies. Given the high rate of initially missed fractures, in conventional radiographs the diagnostic workflow in the clinical setting should emphasize the necessity of early CT imaging in patients with persistent pelvic pain and suspected fractures. In accordance with these findings, Böhme et al. found that since the introduction of standardized CT imaging in patients over 65 years of age with anterior pelvic ring fractures in radiographs or lumbosacral pain, the results of CT scanning had a direct influence on the treatment algorithm [38]. Furthermore, the diagnostic value of sacroiliac joint pain as an indicator of posterior pelvic ring fractures suggests that targeted imaging strategies could improve early diagnosis and management.

The fracture morphology and stability, pain level and mobility are factors that influence treatment recommendation. Conservative treatment is recommended for fractures that are classified as stable [39]. Early mobilization plays a crucial role in preventing complications such as pneumonia, deep vein thrombosis and pressure ulcers [5], 40]. Interventional/surgical treatment is recommended for persistent pain, unstable or displaced fractures or failure of conservative treatment and impeded mobilization [39]. The treatment options for these cases involve interventional methods such as sarcoplasty, as well as various fixation methods such as percutaneous screw osteosynthesis, open procedures such as plate osteosynthesis, and internal or external fixators [41]. Surgical interventions are primarily focused on the management of fractures within the posterior pelvic ring, with the goal of pain relief [42]. The significance of the fixation of the anterior pelvic ring remains unclear, since, in contrast to the results of the clinical studies, biomechanical studies show advantages in terms of stability and fracture progression [43]. Moreover, rigid stabilization constructs such as triangular stabilization may lead to mechanical failure of the osteosynthesis, particularly in the context of poor bone quality, as observed in spine surgery [44]. Furthermore, risk factors such as osteoporosis or sarcopenia should be treated [20].

The OF Pelvis Score has emerged as a promising tool for guiding clinical decision-making and aligning treatment strategies with fracture severity and patient condition. Its high concordance rate with actual treatment decisions suggests that it may be a valuable addition to routine clinical practice. Scores such as the OF Pelvis Score have the capability to inform the decision regarding whether surgical treatment would be beneficial by offering a method of visualising the dynamic clinical presentation of patients with PIF [13]. After validation in a prospective, randomized study this tool could help to avoid longer periods of immobilization, which can lead to significant morbidity [45]. Nevertheless, the score does not provide information about the surgical method to be used.

Concerning pharmaceutical therapy, a discrepancy exists between the literature’s recommendations and clinical treatment of risk factors, such as osteoporosis [46]. The study presented by Novikov et al. found a low rate of PTH treatment initiation for PFIs. The authors attributed this phenomenon to a variety of factors, including the absence of clear data regarding the optimal dosage and duration of treatment, as well as the high cost of the therapy [14]. This highlights that there is still a need for further research regarding the implementation of pharmaceutical therapy to treat underlying diseases such as osteoporosis.

Overall, the treatment of PIF currently still presents a particular challenge, as there is a lack of standardized treatment recommendations that include conservative, surgical and pharmaceutical therapies. There is also a lack of evidence-based recommendations for individualized therapeutic decision-making [32], 47], 48]. The cause is to be found in the heterogeneous data situation, which consists mainly of retrospective studies with small to medium-sized study cohorts, range of outcome parameters and variable study periods [24], 42]. In this context, initiatives such as the SNAPPelvis study of the European Society for Trauma and Emergency Surgery (ESTES) and the S1 guideline of the German Society for Orthopaedics and Trauma Surgery (DGOU) [7] can be considered as approaches to address the existing uncertainty by providing evidence-based recommendations.

Complications associated with PIFs, particularly bleeding risks, remain a topic of debate [15], [16], [17]. While high-energy pelvic fractures are well known to be associated with significant hemorrhage [7], the reviewed studies suggest that low-energy PIFs can also result in considerable blood loss, particularly in patients on anticoagulant therapy. The conflicting findings on bleeding risks necessitate further research to develop clear guidelines for anticoagulation management in patients with PIF.

Finally, the outcomes of patients with PIF highlight the severe impact on mobility, independence, and mortality rates. The reviewed studies confirm a substantial one-year mortality rate of up to more than 20 %, which is comparable to mortality rates seen in hip fractures [49]. It is also demonstrated for hip fractures that the comorbidities are more relevant for the outcome rather than perioperative/surgical factors [50], 51]. It is therefore logical that a special geriatric screening and orthogeriatric co-management is beneficial for patients with PIFs [9]. This also underscores the critical importance of early rehabilitation and secondary fracture prevention strategies. Frailty and pre-injury functional status have emerged as key prognostic factors influencing recovery, further emphasizing the need for individualized treatment plans that address both the fracture and the broader health status of the patient. Here, established scoring systems such as the Charlson Comorbidity Index or ASA-Score can be helpful in assessing the patient’s state of health [52].

The present review is limited in several ways. As a narrative review with a selective search strategy, it does not follow the structured methodology of a systematic or scoping review, including predefined inclusion criteria, quality assessment, or comprehensive reporting according to PRISMA guidelines. The studies included in the review are characterised by heterogeneity in terms of design, sample size, fracture classification used and outcome parameters. This heterogeneity limits the ability to make comparisons and to generalise the findings. Moreover, the majority of studies were retrospective and monocentric, which increases the risk of selection bias and limits the strength of conclusions. Despite these limitations, this review provides a structured overview of current developments and highlights key areas for future research and clinical improvement.

## Conclusions

This review provides a comprehensive overview of recent research on PIF, offering valuable insights into diagnosis, treatment, complications, and outcomes. The findings underscore the importance of an interdisciplinary approach, incorporating early and precise diagnostics, tailored therapeutic strategies, and proactive osteoporosis management to improve patient outcomes. Future studies should focus on refining diagnostic algorithms, optimizing treatment protocols, reveal the effect of systemic treatment of osteoporosis, and evaluating long-term interventions aimed at enhancing recovery and quality of life in this vulnerable patient population.
